# Paediatric high-pitch lung imaging with photon-counting detector computed tomography: a dose reduction phantom study

**DOI:** 10.1007/s00247-025-06235-0

**Published:** 2025-04-15

**Authors:** Michael Zellner, Thomas Sartoretti, Thomas Flohr, Thomas Frauenfelder, Hatem Alkadhi, Christian J. Kellenberger, Victor Mergen

**Affiliations:** 1 Department of Diagnostic Imaging, University Children’s Hospital Zurich, Lenggstrasse 30, Zurich, 8008 Switzerland; 2 Diagnostic and Interventional Radiology, University Hospital Zurich, University Zurich, Zurich, Switzerland

**Keywords:** Paediatric imaging, Paediatric lung, Phantom study, Photon-counting detector, Radiation dose

## Abstract

**Background:**

Photon-counting detector computed tomography (PCD-CT) can reduce radiation dose in paediatric lung imaging.

**Objective:**

The aim of this study was to determine the lowest radiation dose maintaining adequate image quality for high-pitch lung imaging using a PCD-CT in a chest phantom replicating the characteristics of a 5-year-old child.

**Materials and methods:**

The phantom was imaged on a dual-source PCD-CT with five different volume CT dose indices (CTDI_vol_): 0.45 mGy, 0.30 mGy, 0.15 mGy, 0.07 mGy, and 0.01 mGy. Scans were acquired with Sn100 kV in standard and ultra-high resolution modes. Polychromatic images were reconstructed with a 1-mm slice thickness, lung kernel Bl60, without quantum iterative reconstruction and with quantum iterative reconstruction at strengths 2 and 4. Two paediatric radiologists rated reconstructions subjectively, defining adequate image quality as the visibility of small peripheral structures. Objective evaluation included global noise index and global signal-to-noise ratio index.

**Results:**

Exposure times were 0.42 s and 0.84 s for standard and ultra-high resolution modes, respectively. Subjective assessments showed no significant differences across scan modes or quantum iterative reconstruction strengths for both readers at all doses (all, *P* > 0.05). Scans at 0.07 mGy with quantum iterative reconstruction 4 were deemed to maintain adequate image quality at the lowest dose. Global noise index was always lower and global signal-to-noise ratio index always higher in ultra-high resolution compared with standard mode, underscoring noise reduction achieved via ultra-high resolution mode’s small pixel effect.

**Conclusions:**

PCD-CT enables high-pitch lung imaging while maintaining adequate image quality at a radiation dose as low as 0.07 mGy, with quantum iterative reconstruction 4, in a paediatric phantom representing a 5-year-old child.

**Graphical Abstract:**

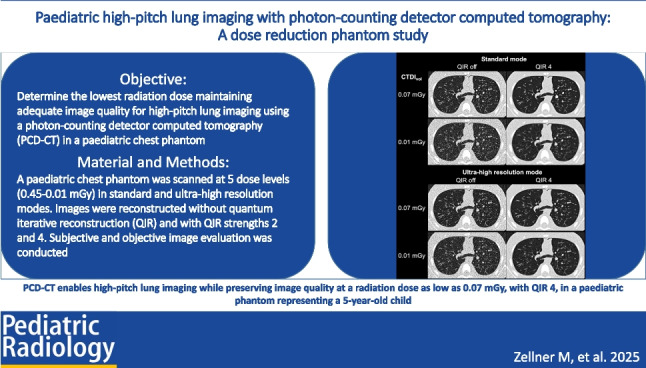

**Supplementary Information:**

The online version contains supplementary material available at 10.1007/s00247-025-06235-0.

## Introduction

Computed tomography (CT) is the reference imaging modality for characterizing airways and lung parenchyma [1]. While advancements in conventional energy-integrating detector CT have introduced various techniques to reduce radiation exposure, the use of CT still entails a considerable radiation burden [2–4]. This concern is particularly pronounced in children, who, due to their elevated metabolic rates and rapid cell proliferation necessary for growth, are more susceptible to radiation-induced effects over time [5–8].

Photon-counting detectors (PCD) are the most recent CT technology characterized by the direct conversion of incoming X-ray photons into electrical signals. PCD-CT offers advantages such as improved geometric dose-efficiency, lower image noise, optimized contrast-to-noise ratio, and inherent spectral information as compared with conventional energy-integrating detector CT [9, 10]. Initial studies using PCD-CT in adult patients demonstrated the feasibility of acquiring chest scans with diagnostic image quality at a radiation dose similar to that of a chest X-ray [11]. Studies evaluating the potential of PCD-CT in paediatric patients remain scarce [6, 12].

When acquiring chest scans in children, patient movement and the inability to follow breath-hold commands pose significant challenges, often necessitating sedation to obtain motion-free, breath-hold chest CTs. Previous studies using the high-pitch mode of dual-source conventional energy-integrating detector CT have demonstrated the feasibility of performing chest CT with diagnostic image quality without anaesthesia. This is mainly attributable to the extremely short acquisition times of such scans of less than a second [13–15].

The aim of this study was to determine the lowest radiation dose maintaining adequate image quality for high-pitch lung imaging using a PCD-CT in a chest phantom replicating the characteristics of a 5-year-old child.

## Material and methods

### Phantom

This study used a paediatric chest phantom (PH- 1 C) measuring 32 cm × 17 cm × 38 cm with a lung density insert (Kyoto Kagaku, Kyoto, Japan). The PH- 1 C phantom is designed to replicate key anatomical structures of the paediatric chest, including the lung, heart, trachea, and surrounding soft tissues. It features a lung density insert with tissue-equivalent materials that mimic the radiographic properties of a 5-year-old child’s lungs providing realistic attenuation and scatter characteristics for CT imaging [16].

### CT acquisition and image reconstruction

The phantom was examined on a first-generation dual-source PCD-CT system (NAEOTOM Alpha, software version VB10; Siemens Healthineers, Forchheim, Germany) equipped with two cadmium telluride detectors. Scans were performed in the high-pitch mode with a pitch factor of 3.2, a gantry rotation time 0.25 s, and at a tube voltage of 100 kV, using tin pre-filtration. The scans were conducted twice, first in the standard mode with a detector collimation of 144 × 0.4 mm, and then in the ultra-high resolution mode with a detector collimation of 120 × 0.2 mm (Table 1). Exposure times during scan acquisition were 0.42 s for the standard mode and 0.84 s for the ultra-high resolution mode. Radiation doses were varied by adjusting the image quality (IQ) level to achieve a volume CT dose index (CTDI_vol_) of 0.45 mGy, 0.30 mGy, 0.15 mGy, and 0.07 mGy, respectively. Additionally, to explore the scanner’s technical limits, the tube current was fixed at the lowest possible value to achieve the minimum radiation dose, resulting in a CTDI_vol_ of 0.01 mGy.

**Table 1:** Scan parameters and radiation dose estimates of the standard and the ultra-high resolution modes

Parameters		Standard mode	UHR mode	
Tube voltage		Sn100 kV	Sn100 kV	
Detector collimation		144 × 0.4 mm	120 × 0.2 mm	
Pitch factor		3.2	3.2	
Table speed		737 mm/s	737 mm/s	
Exposure time		0.42 s	0.84 s	
Scan range		22 cm	22 cm	
Radiation doses				
	CTDI_vol_ (mGy)	DLP (mGy × cm)	SSDE (mGy)	ED (mSv)
1	0.45	10.60	0.90	0.55
2	0.30	6.91	0.59	0.36
3	0.15	3.65	0.31	0.19
4	0.07	1.63	0.14	0.08
5	0.01	0.33	0.03	0.02

*CT* computed tomography, *CTDI*_*vol*_ volume CT dose index, *DLP* dose-length product, *ED* effective dose, *SSDE* size-specific dose estimate, *UHR* ultra-high resolution

**Fig. 1:**
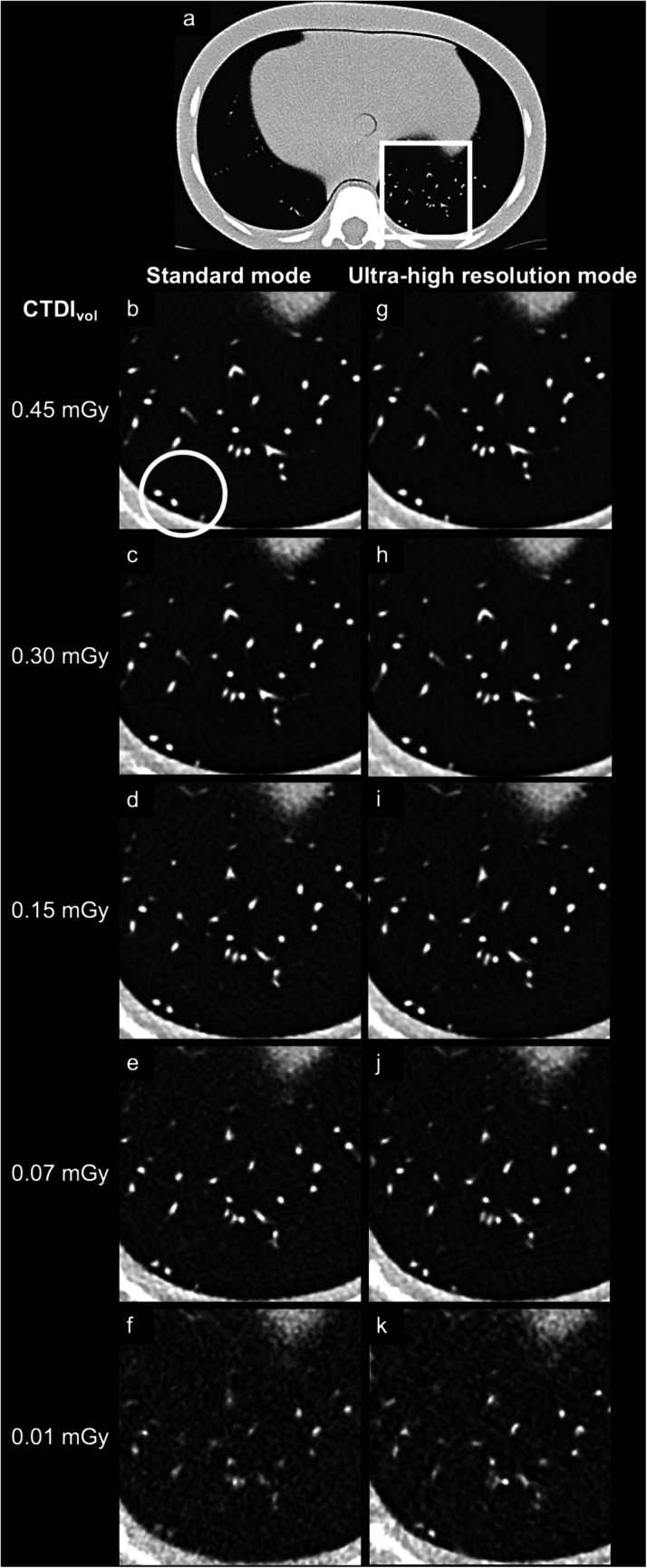
Representative axial images of scans acquired in both standard and ultra-high resolution modes are presented. A *rectangle* highlights the magnified region within the phantom (**a**). The magnified images compare scans from the standard mode (**b**-**f**) and the ultra-high resolution mode (**g**-**k**) across varying radiation doses. All reconstructions were performed using quantum iterative reconstruction at strength 4. Note the worsening visibility of small peripheral structures, such as the three subpleural nodules marked with a *circle*, with a lower radiation dose. *CT* computed tomography, *CTDI*_*vol*_ volume CT dose index

**Fig. 2:**
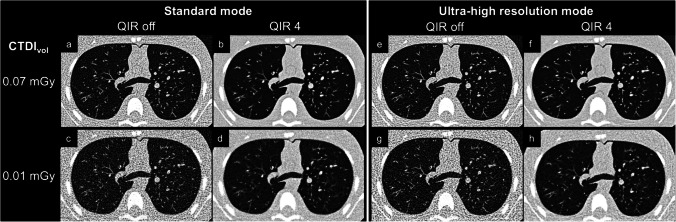
Representative axial images of scans acquired in the standard mode (**a**-**d**) and the ultra-high resolution mode (**e**–**h**) at the two lowest radiation doses (volume CT dose index, CTDI_vol_) of 0.07 mGy (**a**, **b**, **e**, **f**) and 0.01 mGy (**c**, **d**, **g**, **h**). Scans were reconstructed both without using quantum iterative reconstruction (QIR off) (**a**, **c**, **e**, **g**) and with QIR at strength 4 (**b**, **d**, **f**, **h**). Note the considerably reduced image noise and augmented visualization of small peripheral structures in reconstructions with QIR 4 (**b**, **d**, **f**, **h**) compared to those without QIR (**a**, **c**, **e**, **g**). *CT* computed tomography, *CTDI*_*vol*_ volume CT dose index, *QIR* quantum iterative reconstruction

All scans were reconstructed as polychromatic images with a single energy threshold set at 20 keV, given that dual-source ultra-high-resolution scans do not yield energy-resolved data. Slice thickness was set to 1 mm and increment to 0.7 mm, applying the sharp lung kernel Bl60 and using a matrix size of 512 pixels × 512 pixels. All scans were reconstructed without quantum iterative reconstruction (quantum iterative reconstruction off), and with quantum iterative reconstruction at strengths 2 and 4. Figures 1 and 2 depict representative images of the paediatric phantom scans performed at different dose levels and reconstructed with different quantum iterative reconstruction strengths.

### Subjective image evaluation

Two board-certified paediatric radiologists, one with 32 years (reader 1, C.K.) and the other with 11 years (reader 2, M.Z.) of experience in paediatric lung CT, independently evaluated the images. Both readers were blinded to the scanning parameters and radiation doses. Reader 2 (M.Z.) performed the subjective image evaluation twice, with a time interval of 10 weeks. Lung images were assessed on axial image sections with fixed window width of 1500 HU and centre of − 500 HU. Six criteria were investigated: overall image quality, image noise and reticular pattern, presence of streak artefacts, pleural sharpness, sharpness of lung structures, and visibility and detection of small lung structures.

Overall image quality was assessed using a 4-point discrete visual scale, where 1 indicated unacceptable quality (non-diagnostic images), 2 indicated limited quality (sufficient only for restricted clinical interpretation due to high noise), 3 indicated adequate quality (small peripheral structures remained visible), and 4 indicated quality exceeding diagnostic requirements (minimal or no noise). Supplementary material 1 depicts examples with an overall image quality exceeding diagnostic requirement, and an unacceptable, non-diagnostic overall image quality.

Image noise and reticular patterns, as well as streak artefacts, were rated using a 4-point discrete visual scale, where 1 was severe, 2 was moderate, 3 was mild, and 4 was absent. Pleural sharpness represents the clarity of the peripheral lung-to-soft-tissue interface, as well as visibility and detection of small lung structures, where three predefined subpleural nodules in the left lower lobe were evaluated using a 4-point discrete visual scale. Here, 1 corresponded to unacceptable, 2 corresponded to significantly reduced, 3 corresponded to mildly reduced, and 4 corresponded to excellent sharpness or visibility.

### Objective image evaluation

Global noise index and global signal-to-noise ratio index were computed as previously described [10, 17]. In brief, after lung segmentation and extraction of all lung voxels, noise maps were generated slice-wise. The standard deviation of pixel values in the immediate vicinity of a given pixel was calculated to create these noise maps. These noise maps allow for the creation of a histogram representing the noise distribution. Subsequently, the mode value, i.e. the most common noise value, is extracted from the histogram and utilized as the global noise index [18].

To compute the global signal-to-noise ratio index, signal-to-noise ratio maps for the entire lung were first generated. These signal-to-noise maps were obtained by dividing the attenuation by the noise within the target region (i.e. a single pixel and its immediate surrounding). A histogram of the signal-to-noise distribution across the lungs then served to extract the mode value, which was utilized as the global signal-to-noise ratio index [10].

In addition, the mean attenuation of all lung voxels was measured. All steps were performed applying a fully automated computational pipeline in the R programming language.

### Dose estimates

Size-specific dose estimates in milligray (mGy) and effective doses in millisievert (mSv) were calculated using the effective chest diameter combined with size-dependent correction factors obtained from the American Association of Physicists in Medicine [19] and Romanyukha et al. [20]. The effective chest diameter was calculated applying the following formula,

$$\text{Effective diameter}= \sqrt{\text{anteroposterior diameter }\bullet \text{mediolateral diameter}}$$

Effective doses were determined by multiplying the dose-length product (DLP) by a diameter-specific conversion factor of 0.052 [19, 20].

### Statistical analysis

Variables are presented as mean ± standard deviation when normally distributed and as median and interquartile range when non-normally distributed. Categorical variables are reported as counts and percentages. Inter-observer reliability between both readers and intra-observer reliability for reader 2 were evaluated using two-way mixed, average measures intraclass correlation coefficients (ICC [3, *k*]). ICC values below 0.5 indicate a poor, 0.5 to 0.75 a moderate, 0.75 to 0.9 a good, and higher than 0.9 an excellent reliability [21]. Differences of quantitative metrics were investigated by Wilcoxon signed-rank tests. *P*-values were adjusted with the Benjamini–Hochberg procedure for multiple comparisons. A two-tailed *P*-value less than 0.05 was considered to indicate statistical significance. Analyses were performed using SPSS Statistics version 26 (IBM Corp., Armonk, NY).

## Results

### Subjective image quality

Inter-observer reliability was excellent across all six image criteria with an ICC of 0.932 (95% confidence interval, 0.89–0.96). Intra-observer reliability was also excellent for all six image criteria with an ICC of 0.986 (95% confidence interval, 0.91–0.99).

**Fig. 3:**
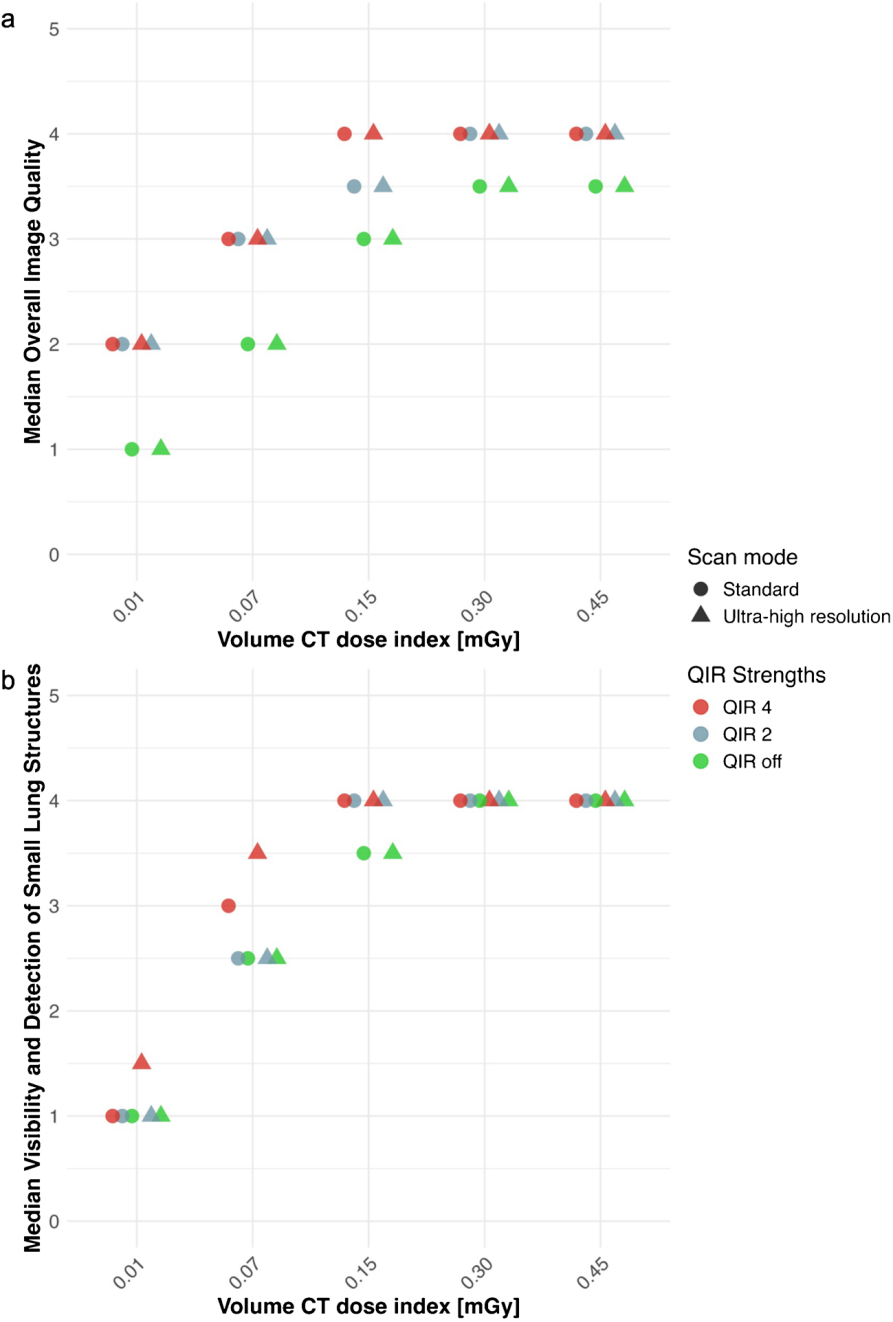
**a**, **b** Scatter plots illustrating the median overall image quality (**a**) and median visibility and detection of small lung structures (**b**) across radiation doses and quantum iterative reconstruction (QIR) strengths, comparing standard and ultra-high resolution modes. *CT* computed tomography, *QIR* quantum iterative reconstruction

Overall image quality was rated as at least adequate by both readers for all reconstructions at the radiation doses (i.e. CTDI_vol_) of 0.45 mGy, 0.30 mGy, or 0.15 mGy, as well as at a radiation dose of 0.07 mGy when using reconstructions with quantum iterative reconstruction 2 or 4 (Fig. 3). Considering both the standard and ultra-high resolution scans, overall image quality did not significantly differ across quantum iterative reconstruction strengths for both readers (all, *P* > 0.05). Similarly, scores of the image noise and reticular pattern did not significantly differ across quantum iterative reconstruction strengths for both readers and both scan modes (all, *P* > 0.05).

Subjective image quality criteria did not significantly differ between standard and ultra-high resolution scans (all, *P* > 0.05). Considering scans at a radiation dose of 0.07 mGy, ultra-high resolution scans reconstructed with quantum iterative reconstruction 4 yielded a slightly improved visibility of structures (3.5 vs. 3.0) compared with standard scans (Fig. 3). Table 2 and Supplementary Material 2 detail the subjective image evaluation results.

**Table 2:**
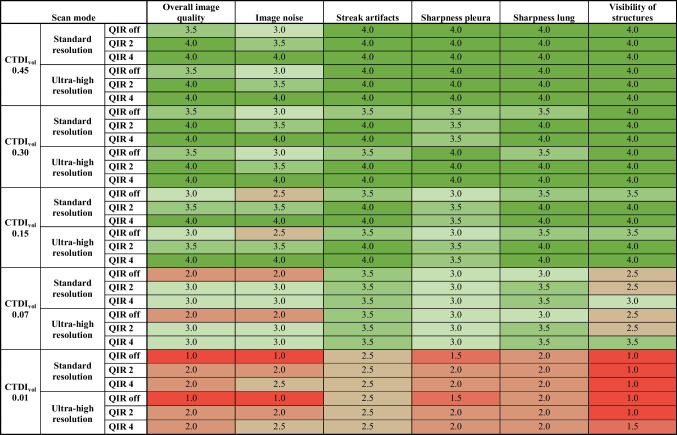
Overview of the results from qualitative analysis for different radiation doses, scan modes, and quantum iterative reconstruction strengths

Qualitative data is presented as the median rating from both readers, with color-coding ranging from reddish (non-diagnostic) to dark green (higher than needed quality). *CT* computed tomography, *CTDI*_*vol*_ volume CT dose index, *QIR* quantum iterative reconstruction

### Objective image evaluation

**Fig. 4:**
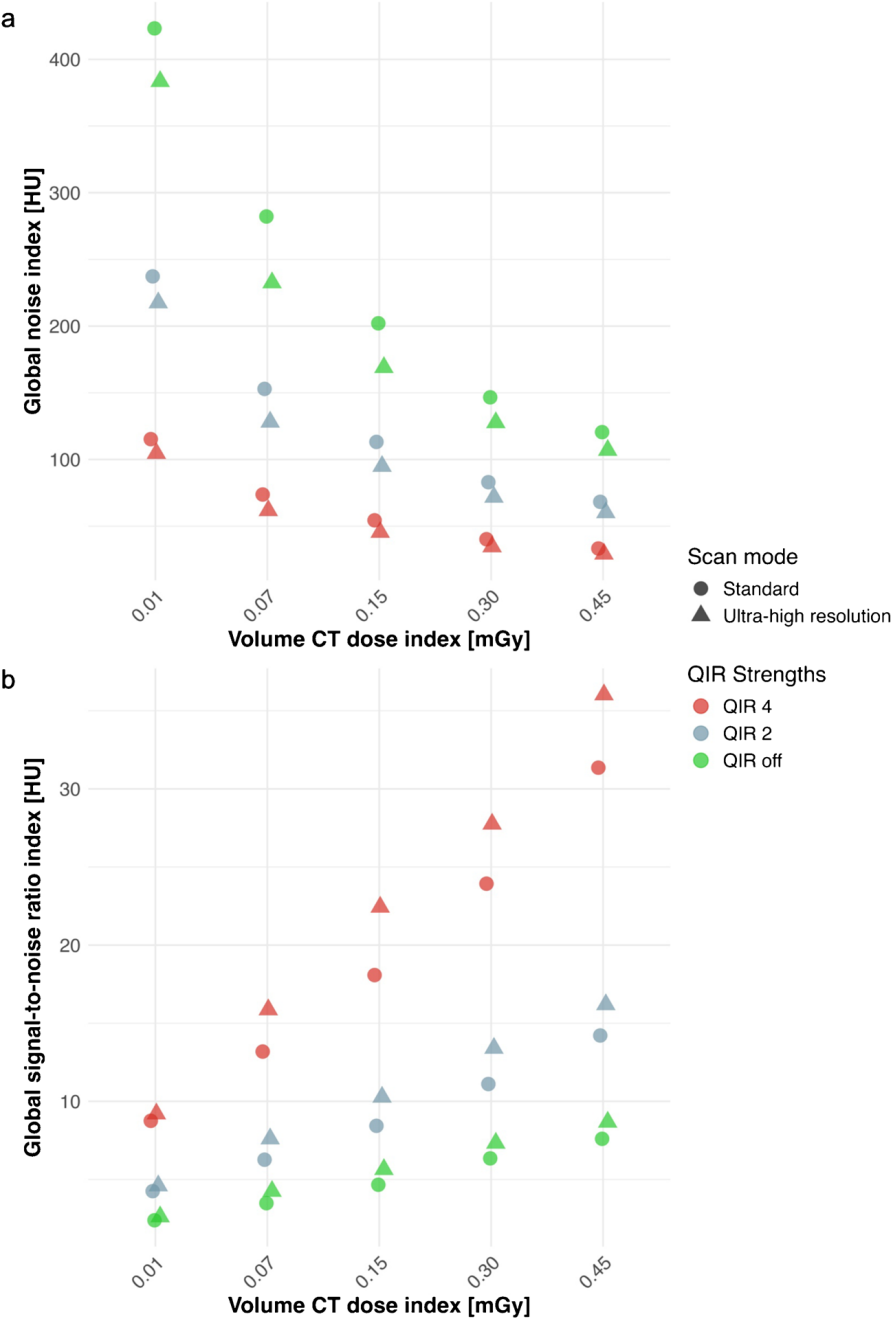
**a**, **b** Scatter plots of the global noise index (**a**) and global signal-to-noise ratio index (**b**) according to the different radiation doses, scan modes, and quantum iterative reconstruction (QIR) strengths. *CT* computed tomography, *QIR* quantum iterative reconstruction

In both standard and ultra-high resolution scans, global noise index was highest at the minimal radiation dose of 0.01 mGy with quantum iterative reconstruction off (423 HU and 383 HU for the standard and ultra-high resolution scan, respectively) and lowest at a radiation dose of 0.45 mGy with quantum iterative reconstruction 4 (33 HU and 29 HU, respectively) (Table 3). Global noise index of ultra-high resolution scans was always lower than the global noise index of standard scans irrespective of the radiation dose and applied quantum iterative reconstruction strength. Higher quantum iterative reconstruction strengths led to lower global noise index for both standard and ultra-high resolution scans, irrespective of the radiation dose. Specifically, global noise index decreased by 72.9 ± 0.6% for standard scans and by 73.0 ± 0.3% for ultra-high resolution scans from quantum iterative reconstruction off to quantum iterative reconstruction 4 (Fig. 4).

**Table 3:** Overview of the results from quantitative analysis for different quantum iterative reconstruction levels

CTDI_vol_ (mGy)	QIR	Global noise index (HU)		Global signal-to-noise ratio index	
		Standard mode	UHR mode	Standard mode	UHR mode
**0.45**	**Off**	120.5	106.9	7.6	8.7
	**2**	68.2	60.2	14.2	16.2
	**4**	33.3	29.1	31.4	36.0
**0.30**	**Off**	146.6	127.7	6.4	7.3
	**2**	83.0	71.8	11.1	13.4
	**4**	40.2	34.6	23.9	27.8
**0.15**	**Off**	202.1	169.0	4.7	5.6
	**2**	113.15	94.9	8.4	10.3
	**4**	54.37	45.5	18.1	22.4
**0.07**	**Off**	282.2	232.5	3.5	4.3
	**2**	152.9	128.1	6.3	7.6
	**4**	73.7	61.7	13.2	15.9
**0.01**	**Off**	423.2	383.5	2.4	2.6
	**2**	237.3	217.5	4.3	4.6
	**4**	115.2	104.6	8.8	9.2

*CT* computed tomography, *CTDI*_*vol*_ volume CT dose index, *QIR* quantum iterative reconstruction, *UHR* ultra-high resolution

Global signal-to-noise ratio index was lowest at a radiation dose of 0.01 mGy with quantum iterative reconstruction off (2.4 and 2.6 for the standard and ultra-high resolution scans, respectively) and highest at a radiation dose of 0.45 mGy with quantum iterative reconstruction 4 (31.4 and 36.0 for the standard and ultra-high resolution scans, respectively). Global signal-to-noise ratio index of ultra-high resolution scans was always higher than global signal-to-noise ratio index of standard scans irrespective of the radiation dose or applied quantum iterative reconstruction strength (Fig. 4). Higher quantum iterative reconstruction strengths led to higher global signal-to-noise ratio index for both standard and ultra-high resolution scans, irrespective of the radiation dose. Applying quantum iterative reconstruction 4, standard scans achieved 285 ± 17% higher global signal-to-noise ratio index and ultra-high resolution scans achieved 283 ± 24% higher global signal-to-noise ratio index compared with quantum iterative reconstruction off.

## Discussion

Radiation protection is crucial in paediatric imaging with children being the most vulnerable patient population. In this phantom study, we aimed to determine the lowest CT dose with maintained adequate image quality for lung imaging with high-pitch PCD-CT. Scans were performed at five different dose levels with the standard detector collimation as well as the ultra-high resolution detector collimation using a paediatric chest phantom mimicking the thorax of a 5-year-old child. Based on our results, adequate image quality, defined as maintaining the visibility of small peripheral structures, was still achieved with a radiation dose as low as CTDI_vol_ of 0.07 mGy, corresponding to size-specific dose estimates of 0.14 mGy and an effective dose of 0.08 mSv.

Current US diagnostic reference values provide a size-specific dose estimates of 3.0 mGy for a thoracic CT without contrast in children, which is 20 times higher than the dose reported in our study [22]. Recently, Tsiflikas et al. indicated that ultra-low-dose chest CT examinations were feasible in paediatric patients with a mean age of 2.6 ± 1.8 years using a PCD-CT at size-specific dose estimates of 0.45 mGy [12]. The patients in the study by Tsiflikas et al. were younger than our chest phantom simulating a 5-year-old child, suggesting an even greater potential for dose reduction [12]. In their study, authors used a pitch factor of 2.4 and did not precise the used detector collimation; however, we assume that they used the standard detector collimation of 144 × 0.4 mm. Interestingly, Zellner et al. 2025 conducted a similar phantom study utilizing a conventional energy-integrating detector CT with a wide detector operating in sequential mode [23]. They reported that images remained diagnostic at radiation doses as low as CTDI_vol_ of 0.13 mGy and size-specific dose estimates of 0.19 mSv while setting a scan range of 16 cm [23]. Remarkably, with PCD-CT, diagnostic image quality was achieved at nearly half the radiation dose with a CTDI_vol_ of 0.07 mGy and size-specific dose estimates of 0.14 mGy while setting a scan range of 22 cm.

In the evaluated PCD-CT, two distinct scan modes are available depending on the detector pixel read-out mechanism, precisely the standard and the ultra-high resolution mode. In the standard mode, four adjacent detector pixels are binned together and read out as one, resulting in a detector collimation of 144 × 0.4 mm. In contrast, the ultra-high resolution mode reads out detector pixels individually, resulting in a detector collimation of 120 × 0.2 mm and finer sampling in the fan direction (scan plane). Interestingly, for fixed slice thickness (1 mm) and kernel (Bl60), global noise index was consistently lower, and global signal-to-noise ratio index consistently higher, when using the ultra-high resolution mode, regardless of the radiation dose and applied quantum iterative reconstruction strength. These observations align with the findings of Huflage et al. and can be attributed to the small pixel effect of the ultra-high resolution mode [24]. Due to the smaller detector pixel size in the fan direction, the modulation transfer function of the measurement system is improved compared with the standard mode. Therefore, for the same kernel, image noise is lower in the ultra-high resolution mode than in the standard mode. This effect is particularly pronounced when sharp kernels with high spatial frequency components are used, such as the sharp lung kernel in our study [24].

Since cooperation of children to hold their breath during chest CT is mostly limited, we placed high emphasis on fast scan acquisition. Previously, several studies using conventional energy-integrating detector CT indicated the robustness of high-pitch scans for acquiring diagnostic chest scans with no need for controlled ventilation or sedation and at low radiation doses [13–15, 25]. Although the scan acquisition time with the ultra-high resolution mode (0.84 s) was twice as long compared with the standard mode (0.42 s), which could be considered a drawback, the high pitch factor of 3.2 kept the scan time well under 1 s. We believe that these very short acquisition times have the potential to enable ultra-low-dose chest scans for paediatric patients without the need for sedative preparation, thereby improving the practicability and clinical impact of this imaging method. However, it is important to note that high-pitch ultra-high resolution scans can only be reconstructed as polychromatic images, distinguishing a single energy threshold and preventing the reconstruction of virtual monoenergetic images. Consequently, standard scans were reconstructed in the same manner to ensure comparability, although these images could also be reconstructed as virtual monoenergetic images.

Use of the quantum iterative reconstruction algorithm is a powerful post-processing tool for noise reduction in low-dose PCD-CT chest scans [10]. Considering objective results, applying quantum iterative reconstruction 4 reduced image noise up to 73% and increased global signal-to-noise ratio index by up to 285% over reconstructions with quantum iterative reconstruction off. Our subjective results revealed that scans performed at radiation dose of 0.07 mGy in the ultra-high resolution mode and reconstructed with quantum iterative reconstruction 4 maintained adequate image quality at the lowest radiation dose. These settings ensure the visibility of the smallest structures in the subpleural space, such as micronodules and tiny blood vessels. Importantly, it must be acknowledged that depending on the imaging task, scans performed with a CTDI_vol_ of 0.01 mGy, corresponding to an effective dose of only 0.02 mSv, may be sufficient.

This paediatric phantom study highlights the potential of PCD-CT to enable thoracic scan acquisition at very low radiation doses (CTDI_vol_ as low as 0.07 mGy), while advanced quantum iterative reconstruction further improves image quality, which may have significant clinical implications for paediatric imaging. Although subjective image quality criteria showed no significant differences between standard and ultra-high resolution scans, ultra-high resolution scans remain preferable, particularly for visualizing small peripheral lung structures, due to their superior quantitative metrics (lower global noise index and higher global signal-to-noise ratio index). However, in cases where heavy movement or breathing artefacts are expected, the faster scan acquisition in the standard mode may be more reliable for obtaining diagnostic images.

Several limitations merit consideration. First, only a limited range of radiation doses was assessed, and phantom scans were performed only once at each dose. Second, only one type of PCD-CT was used, as it is currently the only scanner available for full clinical use. Third, scans were reconstructed with various quantum iterative reconstruction strengths, but the reconstruction kernel and slice thickness remained fixed. Fourth, while the radiologists were blinded to scanning parameters, their awareness that the images were from a phantom may have influenced their perception of diagnostic acceptability. Fifth, the phantom used in the study was designed to represent the chest of a 5-year-old child, without incorporating other phantom sizes, limiting the applicability of the results to younger or older children. Finally, while phantom studies form the foundation of scan protocol optimization, further patient studies are mandatory to validate findings.

## Conclusion

PCD-CT enables high-pitch lung imaging while maintaining adequate image quality at a radiation dose as low as 0.07 mGy, with quantum iterative reconstruction 4, in a paediatric phantom representing a 5-year-old child. These findings suggest considerable potential for lowering radiation exposure in paediatric imaging and should be validated in future patient studies.

## Supplementary Information

Below is the link to the electronic supplementary material.

Supplementary file1 (DOCX 656 KB)

## Data Availability

No datasets were generated or analysed during the current study.
