# Supplementary material for: Paediatric high-pitch lung imaging with photon-counting detector computed tomography: a dose reduction phantom study
Source: Pediatr Radiol. 2025 Apr 15;55(6):1191–201. doi: 10.1007/s00247-025-06235-0 (PMC12119663; doi:10.1007/s00247-025-06235-0)
Supplement: Supplementary file 1 — Supplementary file1 (DOCX 656 KB) [file 247_2025_6235_MOESM1_ESM.docx]

Supplementary material:


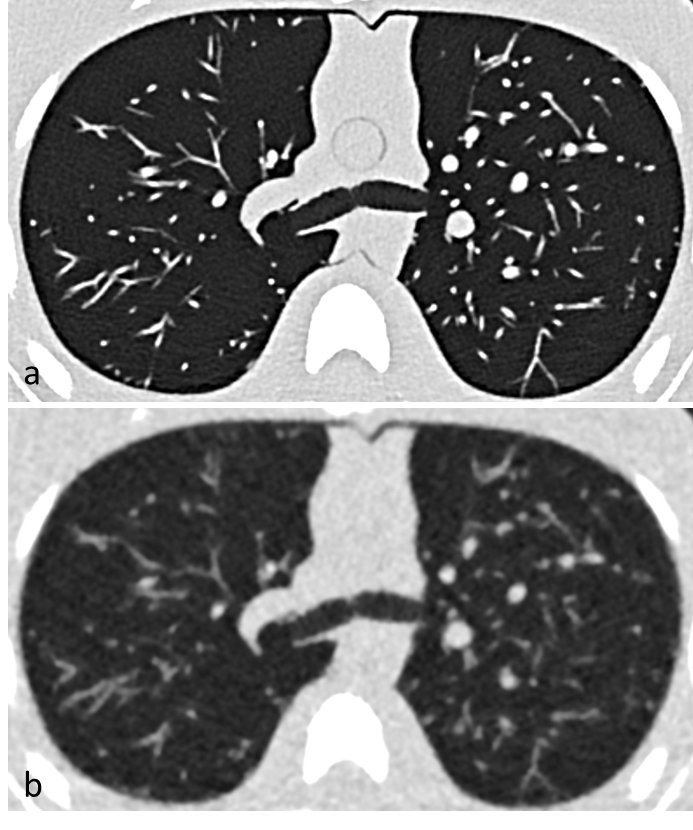


**Supplementary Material 1:** Representative axial images of two scans acquired in ultra-high resolution mode and reconstructed using quantum iterative reconstruction at strength 4: (**a**) scanned at a volume CT dose index of 0.45 mGy, yielding an overall image quality score exceeding diagnostic requirements (minimal or no noise); (**b**) scanned at a volume CT dose index of 0.01 mGy, deemed unacceptable. For detailed quality scores from each reader, refer to **Supplementary Material 2**. *CT* computed tomography

**Supplementary Material 2**: Overview of the results from qualitative analysis for different radiation doses, scan modes and quantum iterative reconstruction strengths for each reader

| Scan mode | | | Overall image quality R1 | Overall image quality R2 | image noise R1 | image noise R2 | streak artefacts R1 | streak artefacts R2 | Sharpness pleura R1 | Sharpness pleura R2 | Sharpness lung R1 | Sharpness lung R2 | Visibility structures R1 | Visibility structures R2 |
| --- | --- | --- | --- | --- | --- | --- | --- | --- | --- | --- | --- | --- | --- | --- |
| CTDI_vol_ 0.45 | Standard resolution | QIR off | 3 | 4 | 3 | 3 | 4 | 4 | 4 | 4 | 4 | 4 | 4 | 4 |
|  |  | QIR 2 | 4 | 4 | 3 | 4 | 4 | 4 | 4 | 4 | 4 | 4 | 4 | 4 |
|  |  | QIR 4 | 4 | 4 | 4 | 4 | 4 | 4 | 4 | 4 | 4 | 4 | 4 | 4 |
|  | Ultra-high resolution | QIR off | 3 | 4 | 3 | 3 | 4 | 4 | 4 | 4 | 4 | 4 | 4 | 4 |
|  |  | QIR 2 | 4 | 4 | 3 | 4 | 4 | 4 | 4 | 4 | 4 | 4 | 4 | 4 |
|  |  | QIR 4 | 4 | 4 | 4 | 4 | 4 | 4 | 4 | 4 | 4 | 4 | 4 | 4 |
| CTDI_vol_ 0.30 | Standard resolution | QIR off | 3 | 4 | 3 | 3 | 4 | 3 | 3 | 4 | 4 | 3 | 4 | 4 |
|  |  | QIR 2 | 4 | 4 | 3 | 4 | 4 | 4 | 3 | 4 | 4 | 4 | 4 | 4 |
|  |  | QIR 4 | 4 | 4 | 4 | 4 | 4 | 4 | 3 | 4 | 4 | 4 | 4 | 4 |
|  | Ultra-high resolution | QIR off | 3 | 4 | 3 | 3 | 4 | 3 | 4 | 4 | 4 | 3 | 4 | 4 |
|  |  | QIR 2 | 4 | 4 | 3 | 4 | 4 | 4 | 4 | 4 | 4 | 4 | 4 | 4 |
|  |  | QIR 4 | 4 | 4 | 4 | 4 | 4 | 4 | 4 | 4 | 4 | 4 | 4 | 4 |
| CTDI_vol_ 0.15 | Standard resolution | QIR off | 3 | 3 | 2 | 3 | 4 | 3 | 3 | 3 | 4 | 3 | 3 | 4 |
|  |  | QIR 2 | 3 | 4 | 3 | 4 | 4 | 4 | 3 | 4 | 4 | 4 | 4 | 4 |
|  |  | QIR 4 | 4 | 4 | 4 | 4 | 4 | 4 | 3 | 4 | 4 | 4 | 4 | 4 |
|  | Ultra-high resolution | QIR off | 3 | 3 | 2 | 3 | 4 | 3 | 3 | 3 | 4 | 3 | 3 | 4 |
|  |  | QIR 2 | 3 | 4 | 3 | 4 | 4 | 4 | 3 | 4 | 4 | 4 | 4 | 4 |
|  |  | QIR 4 | 4 | 4 | 4 | 4 | 4 | 4 | 3 | 4 | 4 | 4 | 4 | 4 |
| CTDI_vol_ 0.07 | Standard resolution | QIR off | 2 | 2 | 2 | 2 | 4 | 3 | 3 | 3 | 3 | 3 | 2 | 3 |
|  |  | QIR 2 | 3 | 3 | 3 | 3 | 4 | 3 | 3 | 3 | 3 | 4 | 2 | 3 |
|  |  | QIR 4 | 3 | 3 | 3 | 3 | 4 | 3 | 3 | 3 | 3 | 4 | 3 | 3 |
|  | Ultra-high resolution | QIR off | 2 | 2 | 2 | 2 | 4 | 3 | 3 | 3 | 3 | 3 | 2 | 3 |
|  |  | QIR 2 | 3 | 3 | 3 | 3 | 4 | 3 | 3 | 3 | 3 | 4 | 2 | 3 |
|  |  | QIR 4 | 3 | 3 | 3 | 3 | 4 | 3 | 3 | 3 | 3 | 4 | 3 | 4 |
| CTDI_vol_ 0.01 | Standard resolution | QIR off | 1 | 1 | 1 | 1 | 3 | 2 | 2 | 1 | 2 | 2 | 1 | 1 |
|  |  | QIR 2 | 2 | 2 | 2 | 2 | 3 | 2 | 2 | 2 | 2 | 2 | 1 | 1 |
|  |  | QIR 4 | 2 | 2 | 3 | 2 | 3 | 2 | 2 | 2 | 2 | 2 | 1 | 1 |
|  | Ultra-high resolution | QIR off | 1 | 1 | 1 | 1 | 3 | 2 | 2 | 1 | 2 | 2 | 1 | 1 |
|  |  | QIR 2 | 2 | 2 | 2 | 2 | 3 | 2 | 2 | 2 | 2 | 2 | 1 | 1 |
|  |  | QIR 4 | 2 | 2 | 3 | 2 | 3 | 2 | 2 | 2 | 2 | 2 | 2 | 1 |

Qualitative data is presented from both readers, reader 1 (R1) and reader 2 (R2). *CT* computed tomography, *CTDI_vol_* volume CT dose index*, QIR* quantum iterative reconstruction
